# Enlarged vestibular aqueduct with bilateral sensorineural hearing loss following cranial trauma: a case report

**DOI:** 10.1016/j.bjorl.2024.101518

**Published:** 2024-10-30

**Authors:** Alexandre Camilotti Gasperin, Bruna Timm Monteiro, Ellen Paula Schetz Zawierucha, Enrico Guido Oliveira Minniti, Matheus Roberto Schetz Alves, Pedro Henrique Vicari Passos

**Affiliations:** a Instituto Paranaense de Otorrinolaringologia (IPO), Curitiba, PR, Brazil; b Pontifícia Universidade Católica do Paraná (PUC-PR), Curitiba, PR, Brazil; c Faculdades Pequeno Príncipe (FPP), Curitiba, PR, Brazil; d Universidade Positivo (UP), Curitiba, PR, Brazil

**Keywords:** Enlarged vestibular aqueduct syndrome, Sensorineural hearing loss, Congenital abnormality, Inner ear

## Introduction

The vestibular aqueduct is a bony compartment formed by the duct and endolymphatic sac, components of the inner ear along with the cochlea and semicircular canals responsible for hearing and balance, respectively.^1^, ^2^, ^3^ This compartment has an average length of 10 mm, with its bony part extending from the medial wall of the vestibule to the posterior surface of the petrous pyramid.^4^, ^5^

Enlarged Vestibular Aqueduct Syndrome (EVAS) is a congenital abnormality, often occurring bilaterally, first described in 1978 by Valvassori and Clemis. Studies suggest that the malformation occurs during the embryological formation of the endolymphatic duct. These alterations may also accompany malformations in the cochlea and semicircular canals.^1^, ^3^, ^4^, ^5^

EVAS predominantly affects females, with a ratio of 3:2, typically manifesting symptoms in childhood and adolescence, being a major cause of hearing loss in the pediatric population. In a minority of cases, it may occur in adults.^1^, ^3^, ^4^

There is a high prevalence of EVAS onset with symptoms originating after cranial trauma, with bilateral Sensorineural Hearing Loss (SNHL) being the most common among them. The generated hearing loss is usually gradual and asymmetrical between the two ears, affecting higher-frequency waves more than lower-frequency ones. Episodes of coordination difficulty, imbalance, and vertigo, especially in adults, are other signs present in EVAS.^1^, ^3^, ^5^

In this study, after the approval of the ethics and research committee, we report the case of a 49-year-old female patient who experienced sudden profound bilateral sensorineural hearing loss following cranial trauma, without prior symptoms.

## Case report

**Fig. 1:**
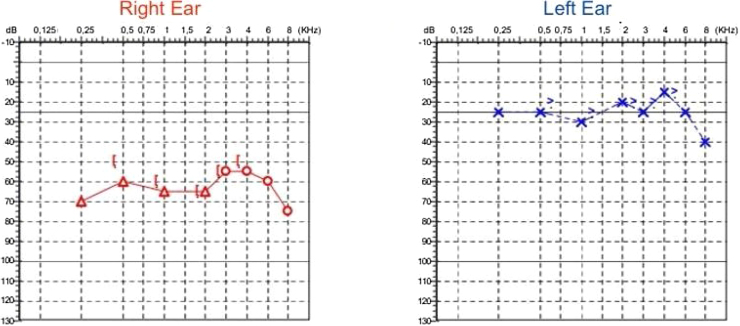
Audiometry showing sensorineural hearing loss, moderately severe in the right ear and mild in the left ear, with an irregular bilateral configuration.

A 49-year-old female patient with no comorbidities presented to an otology referral hospital in Curitiba, Paraná, Brazil. She reported bilateral hearing loss, total in the left ear and partial in the right ear, progressing to rotational dizziness with constant floating sensation, accompanied by nausea and discomfort. Symptoms began after a ground-level fall with direct cranial trauma, impacting the occipital region and resulting in fainting after the progressive myomectomy. The patient denied any prior symptoms. A computed tomography scan at the time of trauma showed no signs of fractures in the inner or middle ear, only a small subgaleal hematoma in the posterior region. Physical examination in the office revealed no abnormalities in otoscopy, oroscopy, rhinoscopy, head, and neck. Audiometry indicated bilateral sensorineural hearing loss, moderate in the right ear with an inverted “U” pattern, and mild high-frequency loss in the left ear (Fig. 1).

The patient was prescribed Prelone for 21 days and Labirin 48 mg, and Magnetic Resonance Imaging (MRI), Brainstem Auditory Evoked Response (BERA), Otoacoustic Emissions (EOAS), Electrocochleography (ECoG), and Vestibular-Evoked Myogenic Potentials (VEMP) were requested.

**Fig. 2:**
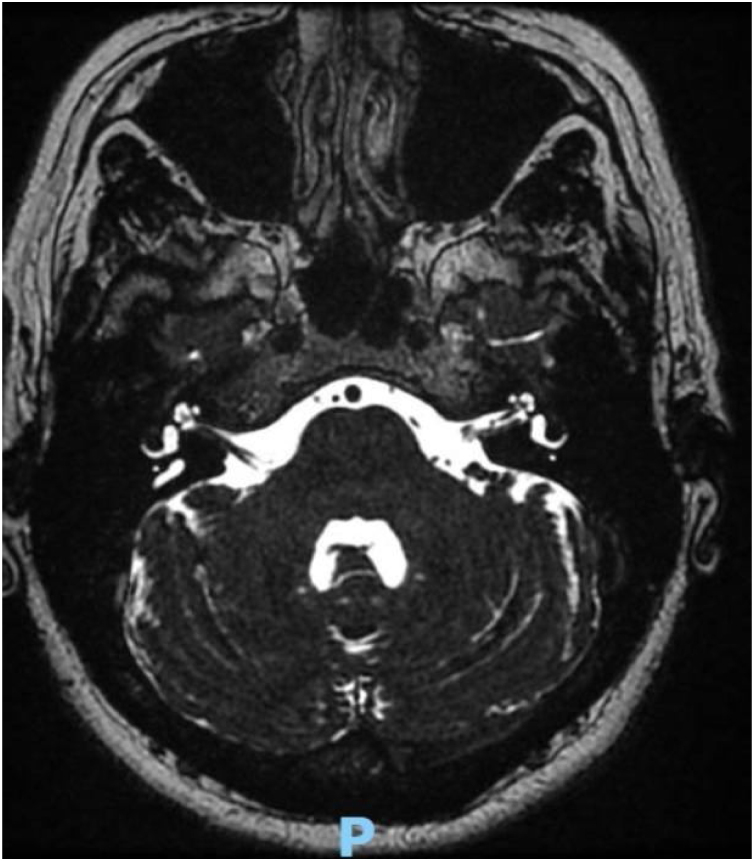
MRI scans: Malformation of the right inner ear, with dilation of the vestibular aqueduct and Alterations that may represent foci of gliosis and/or microangiopathy.

**Fig. 3:**
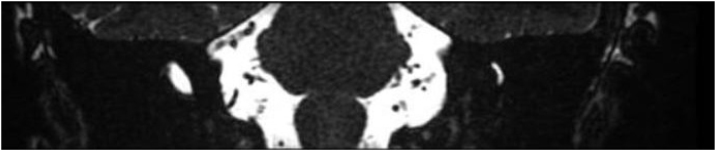
MRI scans: Malformation of the right inner ear, with dilation of the vestibular aqueduct.

On a follow-up visit after 7 days, the patient showed bilateral improvement in hearing without other symptoms. BERA results were unremarkable, EOAS was absent bilaterally, ECoG was absent on the right and 13% on the left, and VEMP showed reduced cervical amplitude, right sided areflexia, and bilateral ocular areflexia. The cranial MRI revealed moderate microangiopathy and severe enlargement of the endolymphatic duct on the right (Fig. 2, Fig. 3).

The diagnosis was congenital alteration of the endolymphatic duct on the right, with asymptomatic progression until the moment of the fall, confirming trauma as the cause of the patient's symptoms, as previously documented in the literature. The treatment plan included a 10-day course of acyclovir, discontinuation of sugar consumption, and completion of the treatment with Prelone and Labirin 72 mg. The patient expressed agreement with all the investigations and actions.

**Fig. 4:**
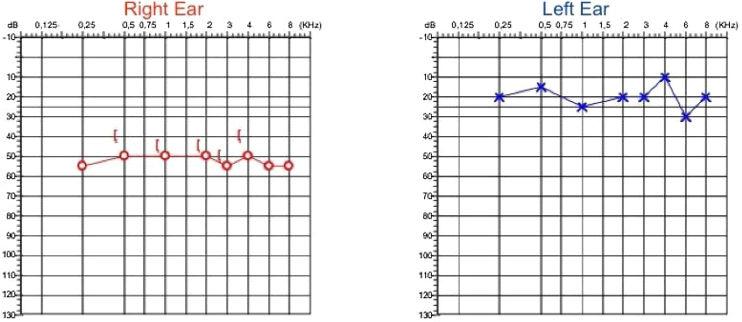
Audiometry showing sensorineural hearing loss, moderately severe in the right ear and mild in the left ear.

On a follow-up visit after 3 months, The patient remained asymptomatic, and a new audiometry showed asymmetric hearing loss of moderate sensorineural type with a horizontal configuration to the right and auditory thresholds within the normal range, with an alteration at 6 kHz (Fig. 4).

## Discussion

Enlarged Vestibular Aqueduct Syndrome (EVAS) is a congenital inner ear abnormality, with studies indicating an association with Pendred syndrome and mutations, primarily in the SLC26A4 (PDS) gene. Additionally, other genes such as the CEVA haplotype, FOXI1, KCNJ10, POU3F4, and possibly GJB2 are implicated in 50 %–60 % of cases of hearing loss and enlargement.^1^, ^6^, ^7^

Among the population most affected by LVAS, females are more commonly affected, with the typical age of symptom onset being childhood and adolescence, although in rare cases it can occur in adults. It is also noted that there is a high prevalence of analyses of this syndrome based on symptoms originating after cranial trauma. In children, LVAS cases are often underreported because these patients with hearing loss, in some instances, do not receive the appropriate radiological referral.

Moreover, even when referred, there can be instances of failure in the analysis of radiological images due to the proximity to the inner ear structures.^2^, ^7^, ^8^, ^9^

The precipitating factors for the syndrome, traumatic brain injury is highlighted, potentially present in 80% of sudden EVAS cases. Other possible causes include barotrauma, upper respiratory tract infections, high fever, sound trauma, and physical exercise.^1^, ^4^, ^7^

Regarding the diagnostic criteria for EVAS, there is no consensus in the literature on the radiographic size for confirmation of enlargement. Initial studies by Valvassori and Clemis suggested a borderline size of 1.5 mm in diameter at the midpoint, while later studies by Jackler and De La Cruz used values greater than 2 mm for diagnosis. In more recent studies in 2007, Vijayasekaran et al. and Boston et al. recommended a new definition, with a diameter greater than 1–1.25 mm.^1^, ^3^, ^7^, ^8^

Magnetic Resonance Imaging (MRI) has proven to be a crucial tool in the diagnosis and management of Enlarged Vestibular Aqueduct Syndrome (EVAS), particularly with the use of Fast Spin-Echo (FSE) pulse sequences. Unlike Computed Tomography (CT), which is limited to the evaluation of the bony structures of the labyrinth, MRI allows for detailed visualization of soft tissues and fluids within the bony labyrinth. This superiority in analysis provides a better understanding of inner ear anomalies, highlighting the dilation of both the endolymphatic duct and sac, which are often present in patients with EVAS. Moreover, MRI is especially useful in differentiating other pathologies that may mimic the symptoms of EVAS, contributing to a more accurate diagnosis and a more targeted therapeutic approach. Furthermore, the role of audiometry is emphasized; although it does not confirm the diagnosis, it is essential for assessing and monitoring the progression of the condition.^4^, ^7^, ^9^

In the treatment of LVAS, therapy with systemic or intratympanic corticosteroids is emphasized, as they have the potential to significantly increase auditory capacity at different frequencies. However, various therapies have been tested to prevent hearing loss, such as endolymphatic sac decompression, diversion of the endolymphatic sac to the subarachnoid space, and hyperbaric oxygen therapy. Nevertheless, all these approaches have not yielded satisfactory results. Given this scenario, cochlear implantation should be considered as a cornerstone of treatment for patients with hearing loss.^4^, ^7^, ^9^

## Conclusion

Enlarged vestibular aqueduct is a major cause of sensorineural hearing loss in children, with its pathophysiological mechanisms remaining uncertain, despite common precipitating factors such as traumatic brain injury. Treatment is based on auditory rehabilitation, and in some cases, cochlear implantation, with corticosteroid therapy showing significant response in some studies, though its results are not yet conclusively established.

## Financing

This research did not receive any specific funding from funding agencies in the public, commercial or not-for-profit sectors.

## Declaration of competing interest

The authors declare no conflicts of interest.
